# Simulating human behavioral changes in livestock production systems during an epidemic: The case of the US beef cattle industry

**DOI:** 10.1371/journal.pone.0253498

**Published:** 2021-06-24

**Authors:** Qihui Yang, Don M. Gruenbacher, Gary L. Brase, Jessica L. Heier Stamm, Scott A. DeLoach, Caterina M. Scoglio

**Affiliations:** 1 Department of Electrical and Computer Engineering, Kansas State University, Manhattan, Kansas, United States of America; 2 Department of Psychological Sciences, Kansas State University, Manhattan, Kansas, United States of America; 3 Department of Industrial and Manufacturing Systems Engineering, Kansas State University, Manhattan, Kansas, United States of America; 4 Department of Computer Science, Kansas State University, Manhattan, Kansas, United States of America; University of Nicolaus Copernicus in Torun, POLAND

## Abstract

Human behavioral change around biosecurity in response to increased awareness of disease risks is a critical factor in modeling animal disease dynamics. Here, biosecurity is referred to as implementing control measures to decrease the chance of animal disease spreading. However, social dynamics are largely ignored in traditional livestock disease models. Not accounting for these dynamics may lead to substantial bias in the predicted epidemic trajectory. In this research, an agent-based model is developed by integrating the human decision-making process into epidemiological processes. We simulate human behavioral change on biosecurity practices following an increase in the regional disease incidence. We apply the model to beef cattle production systems in southwest Kansas, United States, to examine the impact of human behavior factors on a hypothetical foot-and-mouth disease outbreak. The simulation results indicate that heterogeneity of individuals regarding risk attitudes significantly affects the epidemic dynamics, and human-behavior factors need to be considered for improved epidemic forecasting. With the same initial biosecurity status, increasing the percentage of risk-averse producers in the total population using a targeted strategy can more effectively reduce the number of infected producer locations and cattle losses compared to a random strategy. In addition, the reduction in epidemic size caused by the shifting of producers’ risk attitudes towards risk-aversion is heavily dependent on the initial biosecurity level. A comprehensive investigation of the initial biosecurity status is recommended to inform risk communication strategy design.

## 1. Introduction

Industrial livestock production is characterized by intensive and high-throughput systems, with all parts of the chain from birth to slaughter always operating at full capacity. Disruptions that initially occur at one part of the chain can immediately impact both upstream and downstream aspects due to entities’ demand-supply relationships. Such impacts could be amplified and propagated throughout the chain [1, 2]. For instance, the ongoing COVID-19 pandemic has dramatically impacted people’s normal activities and left significant economic consequences to industries, among which the livestock production industries are one the most negatively impacted sectors [3–5]. In the United States, outbreaks identified among meat-processing workers led to the closure of several meat-processing plants, causing sharp disruptions in livestock production. Peel et al. [6] suggested that COVID-19 could result in $13.6 billion economic losses to the US beef industry, highlighting the fragility of today’s intensive livestock production industries against unexpected events.

Fortunately, as livestock production has suffered substantial losses from many prior unexpected events such as foot-and-mouth disease (FMD) outbreaks and swine fever in the past, the number of sophisticated models developed for livestock diseases has surged. However, many of these epidemiological frameworks did not model human behavioral changes during epidemics [7]. A proper understanding of animal disease dynamics depends not only on accurate epidemiological parameter estimates and an appropriate underlying movement network, but also on the disease-related human behavior factors. Not accounting for human behavioral changes in epidemiological models could lead to a substantial bias. Thus, exploring the potential impact of behavioral changes on disease epidemics is vital to shedding light on future disease management [8, 9].

Several projects have applied multiplex networks to study the interplay between human behavior factors and epidemic dynamics [10–13]. The substantial influence of human behavior factors such as risk attitudes has again drawn scholars’ attention during the current global pandemic. Studies have found that more risk-averse attitudes were related to a dramatic decline in human mobility and were significantly related to adherence to the recommended behaviors [14, 15]. In the context of livestock production, biosecurity is referred to as implementing control measures to decrease the chance of animal disease spreading [16]. Several studies have analyzed the factors that are likely to influence farmers’ willingness to comply with biosecurity protocols, and examples of such factors include knowledge of disease prevalence, farmers’ risk perception, and disease experience of neighboring farmers [7, 17, 18]. Mendes et al. [19] emphasized the importance of controlling chronic livestock disease considering farmers’ different responses to the perceived risk. Tago et al. [20] illustrated how farmers’ strategic behavior can considerably reduce the efficacy of movement restriction policy for disease spread in a French cattle trade network.

This study aims at investigating *how human behavioral changes around biosecurity could affect disease transmission dynamics in livestock production systems*. We will apply an agent-based modeling (ABM) approach, which allows simulating interactions among heterogeneous individuals in complex systems. As a bottom-up approach, ABM has been widely applied to diverse areas, including economics, agriculture, transportation, and healthcare [21]. Abdulkareem et al. [22], for example, used an agent-based approach to compare the effects of individual and group learning on the decision-making process regarding risk appraisal and protective decisions. Their results showed that behavioral changes could lead to different epidemic durations and sizes. Bucini et al. [23] found that shifting 37.5% of the producer agents toward a risk-averse position could result in a significantly sharp decrease in the epidemic size of the porcine epidemic diarrhea virus. Sok and Fischer [24] modeled farmers’ vaccination decisions over bluetongue disease, taking risk perception and social pressure into account. Their results suggested that a risk communication strategy has an advantage over financial compensation in increasing vaccination uptake. Regarding beef cattle production, prior ABMs have been designed for disease prediction and control measures [25–29]. Other works have taken the cattle industry as a case study to analyze the risk management strategies in reaction to disease outbreaks, agricultural policy interventions, and market dynamics in social-ecological systems [30–32]. To the best of the authors’ knowledge, few works have examined human behavioral changes on animal disease transmission. This work adds a new element to this domain with a focus on the following perspectives: (i) random or targeted selection in risk communication audiences, and (ii) initial biosecurity status.

In this paper, we will model beef cattle production systems in southwest Kansas, the United States, as a case study and examine the impact of human behavior factors on a hypothetical FMD spreading. FMD could potentially threaten cloven-hoofed animals worldwide, and concerns about its reintroduction into the US have escalated due to increased international trade [33, 34]. It could spread so quickly through cattle herds that a delay of even a few days would be devastating, resulting in losses in both animals and export markets. After a latent period of 1 day to 3 days, the individual cattle exposed to the FMD virus can become infectious and infect other animals [35]. The modeled beef cattle production chain spans from cow-calf ranches to stockers, to feedlots, to packers. We consider disease spreading through direct and indirect contact routes, i.e., movement of infected animals and contaminated fomites. In addition, the model associates risk attitude categories to individuals, including producer agents, packer agents, and fleet companies, and takes the biosecurity level as an indicator to reflect human behavioral change during epidemics. We assume that individuals in the risk-averse category are likely to increase their biosecurity levels more promptly when facing a rise in the regional disease incidence than those with risk-tolerant attitudes. Here, biosecurity level indicates individual’s compliance to biosecurity protocols, which are designed to reduce the risks of introducing disease agents to the premises. For example, a producer with a high biosecurity level will strictly follow the cleaning and disinfection guidelines of vehicles.

Resources for risk communication can be limited; for example, lack of employees may exist during the crisis. Therefore, it is necessary to design an effective risk communication strategy to protect livestock production. In scenario analyses, we change the percentage of producers in the risk-tolerant category in the total producer population under both random and targeted selection strategies. In the random strategy, producers are randomly selected as risk-averse or risk-tolerant. In the targeted strategy, producers are selected as risk-averse in descending order of their cattle capacity and the rest are assigned as risk-tolerant. In other words, risk communication resources will be allocated to large producers first, given the limited resources. In addition, we explore the impact of individuals’ initial biosecurity levels on the epidemic dynamics. The outcomes are expected to deepen our understanding of the link between disease transmission dynamics and human behavioral changes, and improve disease management in livestock production, from on-farm management to the transportation of animals.

## 2. Materials and methods

In this section, we present details about the agent-based model added with the human behavior component.

### 2.1 Model description

**Fig 1:**
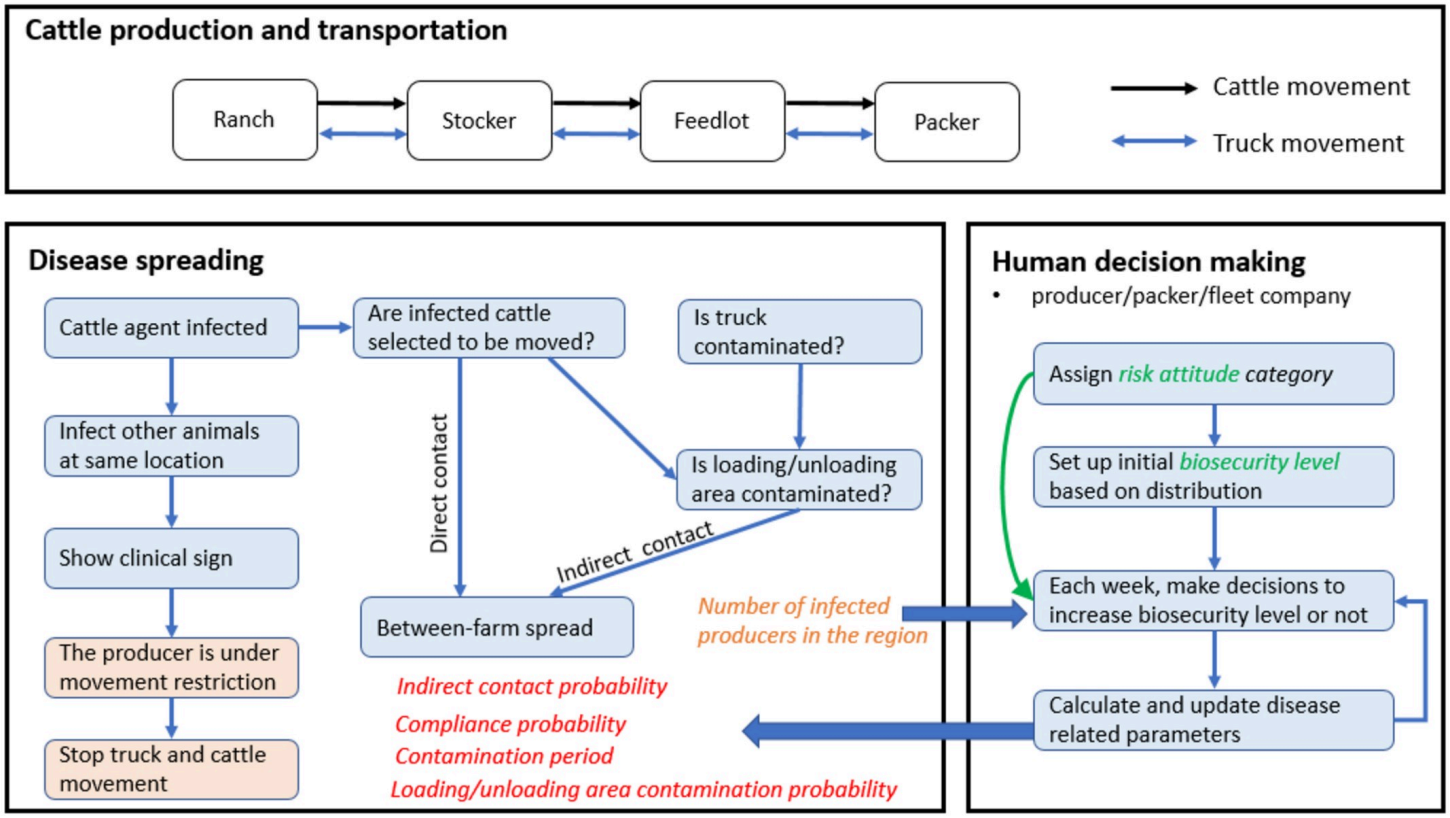
Model structure.

The model was developed in AnyLogic software and built on top of the agent-based model in Yang et al. [36, 37], which simulates the FMD spreading in the cattle production system. The added features integrated the human behavioral changes around biosecurity with the epidemic spreading process. The model structure is shown in Fig 1.

The model explicitly simulates business operations of the beef cattle industry and the associated transportation services on a GIS map. Cattle are sold and transported between cattle producers, including cow-calf ranches, stockers, and feedlots. In spring, cows produce a new generation of calves that are fed until around 450 pounds and are then sold to stockers. Cattle raised in stockers are sold to feedlots at about 650 pounds. Heifers and steers in feedlots are moved to packers at approximately 1250 pounds and 1350 pounds, respectively. At each packer, on average, 6000 head of cattle enter the meat-production process per day. Entry point agents, placed on the system boundaries, represent cattle coming from outside southwest Kansas. The packer, producer, and fleet (truck company) agents all possess and dispatch vehicles to transport cattle among premises on the GIS map roads.

For a hypothetical FMD outbreak, we simulate disease transmission through the movement of infected cattle (direct contact) and the movement of contaminated fomites (indirect contact). Each cattle agent may transition among states of susceptible, exposed, infectious, and removed. Each truck follows a clean-infected-clean cycle and may become contaminated by visiting a fomite-infected location on its route. Contaminated trucks may further spread the infection to subsequent locations visited. A producer agent will transition to the infected state once it has infectious cattle. Then, after a total time from infection to starting control measures, the producer agent will depopulate all its cattle and follow movement restrictions. For the sake of computational efficiency, a scaling factor of 15 is used for parameters related to the number of cattle such that one cattle agent represents fifteen physical animals.

Information about the study population and parameters is shown in Table 1. The producer agent includes three categories, namely ranches, stockers, and feedlots.

**Table 1:** Model parameters.

Parameters	Value	References
***Initial network parameters***		
Total producers (*N*)	301	Yang et al. [36]
Number of ranches	18 (5.98%)	
Number of stockers	50 (16.61%)	
Number of feedlots	233 (77.41%)	
Total cattle inventory at the beginning of the simulation	2,913,007	
Number of cattle at ranches	14,050	
Number of cattle at stockers	79,768	
Number of cattle at feedlots	2,819,189	
***Disease parameters***		
Probability of transmission when an infected cattle agent contacts a susceptible cattle agent	0.95	Boklund et al. [38]
Length of cattle latent period (days) [Pert distribution]	Pert(1.2, 1.2, 2.4)	Yadav et al. [35]
Length of cattle infectious period (days) [Normal distribution]	Normal(11.4, 1.1)	Yadav et al. [35]
Total time from infection to beginning control measures for the first FMD case (days)[Triangular distribution]	*μ* = 8.6; 6.0 ≤ *x* ≤ 12.8	Walz et al. [39]
Total time from infection to beginning control measures for subsequent FMD cases [Triangular distribution]	*μ* = 6.6; 4.5 ≤ *x* ≤ 10.5	Walz et al. [39]
Contamination period *H*_*base*_ for a truck or a loading/unloading area of premises (days)	14	Rossi et al. [40]
Length of packer infection period (days) [Triangular distribution]	*μ* = 5; 0 ≤ *x* ≤ 10	Wiltshire [41]
Indirect transmission probability *P*_*ind_base*_	0.35	Bucini et al. [23]
Probability that infected cattle will contaminate packer/producer receiving area *P*_*inLoad_base*_	0.75	Wiltshire [41]
Probability to comply with movement restrictions *P*_*mr_base*_	1.0	Assumed

### 2.2 The human decision-making process

In the model, we incorporate the feature of risk attitude into each individual to influence its decision to increase biosecurity level, thereby indirectly affecting the disease transmission in the system. We consider two risk attitude categories, namely risk-averse and risk-tolerant, which will influence individuals’ compliance with the biosecurity protocols.

As shown in Fig 1, each individual *i* is associated with variables of *risk attitude category Att*^*i*^ and biosecurity level ${bs}_{t}^{i}$. First, we initialize the value of *Att*^*i*^ for individual *i*, which will remain unchanged during the simulation. Then, for each type of the agent population (producer/packer/fleet agent), we set the initial *biosecurity level* (${bs}_{t_{0}}^{i}$) for individual *i* according to the assumed distributions. The biosecurity level ${bs}_{t}^{i}$ will change over time.

Each week, individual *i* makes a decision on whether to increase its biosecurity level based on the following equation according to Bucini et al. [23]:

$${bs}_{t}^{i}={bs}_{t-1}^{i}+\Delta bs∙\psi \left({Att}^{i},{NI}_{t}\right),$$ (1)

where the biosecurity increase Δ*bs* is a constant value and is assumed to be 2.5; $\psi \left(Att^{i},NI_{t}\right)=\left\{\begin{array}{ll} 0, & \text{if}\;s>P_{increase}\\ 1, & \text{if}\;s<P_{increase} \end{array}\right.,$ *s* ∈ *U*(0, 1); and ${bs}_{t}^{i}$ is assumed to range between [0, 8]. *P*_*increase*_ is the probability of increasing biosecurity level and is calculated by Eq (2).

$$P_{increase}=\frac{1}{1+e^{m\left({NI}_{t}-rI_{0}\right)}}$$ (2)

**Fig 2:**
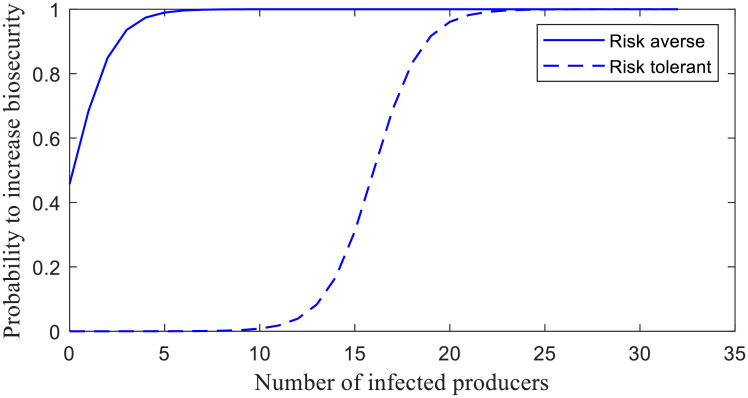
Probability to increase biosecurity as a function of the number of infected producers driven by risk attitude.

*NI*_*t*_ equals the number of infected producers in southwest Kansas at time *t*; *m* = −0.95 and *rI*_0_ = 0.18 for a risk-averse category; and *m* = −0.8 and *rI*_0_ = 16 for a risk-tolerant category. The relationship between *P*_*increase*_ and *NI*_*t*_ (Eq (2)) is presented in Fig 2. It shows how the increase in biosecurity level ${bs}_{t}^{i}$ is influenced by the regional disease prevalence and an individual’s risk attitude. As the number of infected producers increases, risk-averse individuals may be more prompt to make changes in the biosecurity practices than the risk-tolerant ones.

The model uses the biosecurity level ${bs}_{t}^{i}$ in logistic functions (Eq (3)) to calculate the human-behavior related disease parameters, including indirect transmission probability $P_{ind}^{i}$, the contamination period *H*^*i*^, compliance probability with movement restrictions $P_{mr}^{i}$, and the probability that infected cattle will contaminate the packer/producer receiving area $P_{inLoad}^{i}$.


$$\left\{\begin{array}{c} \begin{array}{c} P_{ind}^{i}=P_{ind\_base}\frac{1}{1+e^{m\left(bs_{t}^{i}-bs_{0}\right)}}\\ H^{i}=H_{base}\frac{1}{1+e^{m\left(bs_{t}^{i}-bs_{0}\right)}} \end{array}\\ \begin{array}{c} P_{mr}^{i}=P_{mr\_base}\frac{1}{1+e^{m\left(bs_{t}^{i}-bs_{0}\right)}}\\ P_{inLoad}^{i}=P_{inLoad\_base}\frac{1}{1+e^{m\left(bs_{t}^{i}-bs_{0}\right)}} \end{array} \end{array}\right.$$ (3)

In Eq (3), *bs*_0_ = 4.0 and *m* = 1.3 according to Bucini et al. [23]. Values of *P*_*ind*_*base*_, *H*_*base*_, *P*_*mr*_*base*_, *P*_*inload_base*_ are set according to Table 1.

From Eq (3), a high biosecurity level will result in a low infection likelihood, indicating the high effectiveness of biosecurity measures. For example, vehicles can be cleaned and disinfected more frequently between subsequent shipments, leading to a shorter contamination period *H*^*i*^. FMD outbreaks would lead to movement restrictions in the region. Individuals of higher biosecurity level will stop all movement of animals and vehicles with a higher compliance probability $P_{mr}^{i}$. Producers can invest facilities and equipment for washing and disinfecting vehicles entering the managed premises, which may lead to a smaller value of $P_{ind}^{i}$. Cattle loading/unloading areas should be kept clean, suggesting a lower $P_{inLoad}^{i}$.

Location-specific values in the disease parameters are assigned following Table 2. For example, if location A is fomite-infected, the probability that the truck will be infected when the truck arrives at location A to begin a shipment from location A to location B will be $P_{ind}^{A}$. If location B is fomite-infected, the probability that the truck will be infected when the truck arrives at location B will be $P_{ind}^{B}$. If the truck transporting cattle between location A and location B is contaminated, the probability that location A will be fomite-infected due to the arrival of the contaminated truck will be $P_{ind}^{A}$.

**Table 2:** Location-specific value in the disease parameters.

Parameter	Description	Interaction	Location *i*
$P_{ind}^{i}$	The probability that the truck will be infected if the producer/packer is fomite-infected	Cattle shipment between producer A→producer/packer B	
	The probability that producer/packer will be fomite-infected if the arriving truck is contaminated	(1) Truck arrives at A	(1) A
		(2) Truck arrives at B	(2) B
*H*^*i*^	The contamination period of trucks	-	Truck owner (Producer/Packer/Fleet)
	The contamination period of the producer loading/unloading area		Producer
	The contamination period of the packer receiving area		Packer
$P_{inLoad}^{i}$	The probability that producer/packer will be fomite-infected if there are infectious cattle loaded/unloaded	Cattle shipment between producer A→producer/packer B	
		(1) Loading cattle at A	(1) A
		(2) Unloading cattle at B	(2) B
$P_{mr}^{i}$	Compliance probability	-	Producer

### 2.3 Scenario analysis

During initialization, *α* percentage of the packer/fleet population are randomly selected as risk-averse (*Att*^*i*^ = 0), and the rest are characterized as risk-tolerant (*Att*^*i*^ = 1). Within each risk attitude category, we set up the initial biosecurity value (${bs}_{t_{0}}^{i}$) for each packer/fleet agent *i* according to the following initial biosecurity distribution: 50% of the total population with initial biosecurity ${bs}_{t_{0}}^{i}=0$, 40% with ${bs}_{t_{0}}^{i}=2.7$, 10% with ${bs}_{t_{0}}^{i}=5.3$, 0% with ${bs}_{t_{0}}^{i}=8$ [23].

In Table 3, three simulation sets are designed based on different combinations of two factors in the producer population: (a) random or targeted selection strategy for risk-averse producers, and (b) distribution of initial biosecurity levels. In the targeted selection strategy, we progressively select producers to be risk-averse, one after another, in the decreasing order of their cattle capacity, and the rest will be set as risk-tolerant. In the random selection strategy, producers are randomly selected as risk-averse or risk-tolerant.

**Table 3:** Description of the simulation set.

Simulation set	Risk-averse producer selection	Initial biosecurity distribution
I	Random strategy	${bs}_{t_{0}}$ for risk-averse category: [0, 2.7, 5.3, 8]—[50%, 40%, 10%, 0]
		${bs}_{t_{0}}$ for risk-tolerant category: [0, 2.7, 5.3, 8]—[50%, 40%, 10%, 0]
II	Random strategy	${bs}_{t_{0}}$ for risk-averse category: [0, 2.7, 5.3, 8]—[0, 40%, 10%, 50%]
		${bs}_{t_{0}}$ for risk-tolerant category: [0, 2.7, 5.3, 8]—[0, 40%, 10%, 50%]
III	Targeted strategy	${bs}_{t_{0}}$ for risk-averse category: [0, 2.7, 5.3, 8]—[50%, 40%, 10%, 0]
		${bs}_{t_{0}}$ for risk-tolerant category: [0, 2.7, 5.3, 8]—[50%, 40%, 10%, 0]

In simulation sets I and III, producers within each risk category follow the same distribution as packers and fleet companies. In simulation set II, the highest biosecurity levels are assigned to 50% of the producers. Regarding risk category selection, we randomly select risk-averse producers in simulation sets I and II but perform a targeted strategy in simulation set III.

Within each simulation set, each scenario represents a different proportion of risk-averse producer agents in the total producer population (*α*) varied between 0 and 100% with an interval of 20%. We record the number of infected producers/packers and the number of infected cattle over time for each simulation. We perform 1000 simulation runs for each scenario, and the simulation period for each run is 200 days.

## 3. Results and discussion

This section presents and compares the simulation results among the three simulation sets to evaluate the impact of risk attitudes on epidemic dynamics.

**Fig 3:**
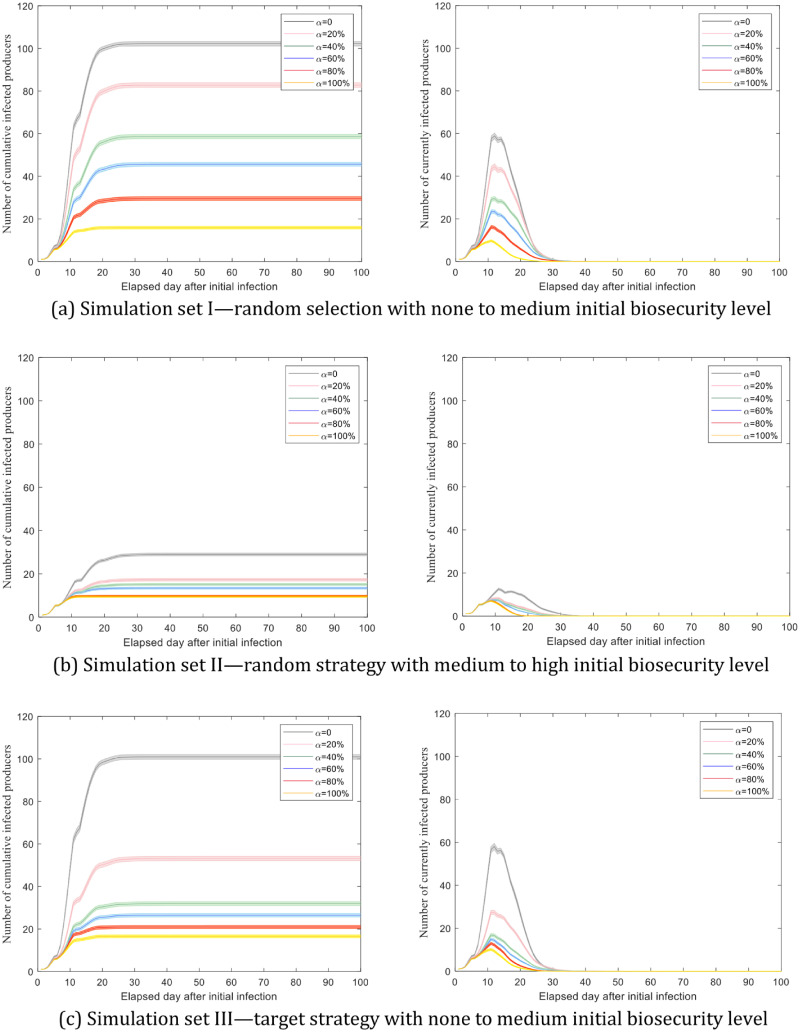
The number of cumulative infected and the number of currently infected producers over time. Simulation results are averaged over 1000 runs and associated with 95% confidence intervals.

Fig 3 shows the average number of cumulative infected producers and the number of currently infected producers over time with 95 percent confidence intervals. In Fig 3(a), as the percentage of risk-averse producer agents (*α*) increases from 0 to 100% with 20% as an interval, the final average number of cumulative infected producers gradually decreases with intervals ranging between 13.01–24.13. In Fig 3(b), 20% of producers in the risk-averse category can achieve a relatively good performance in reducing epidemic size, and after 20%, the epidemic size reduces more slowly.

In Fig 3(c), the final average number of cumulative infected producers reduces sharply from 100.94 to 31.92 as *α* varies between 0–40%, and then decreases gradually with smaller gaps, ranging between 4.39–5.50. It suggests that the system needs at least 40% of the producer population as risk-averse to achieve a significantly steeper decrease in the epidemic size under a targeted strategy. In comparison, the number of cumulative infected producers under a random strategy at *α* = 40% is 58.67 (95% CI: 57.65–59.69) in Fig 3(a).

Similar patterns are observed in the number of currently infected producers in Fig 3 (right panel). In addition, simulation set II has the lowest peak in terms of producer infection as its overall initial biosecurity level is the highest among the three simulation sets.

**Fig 4:**
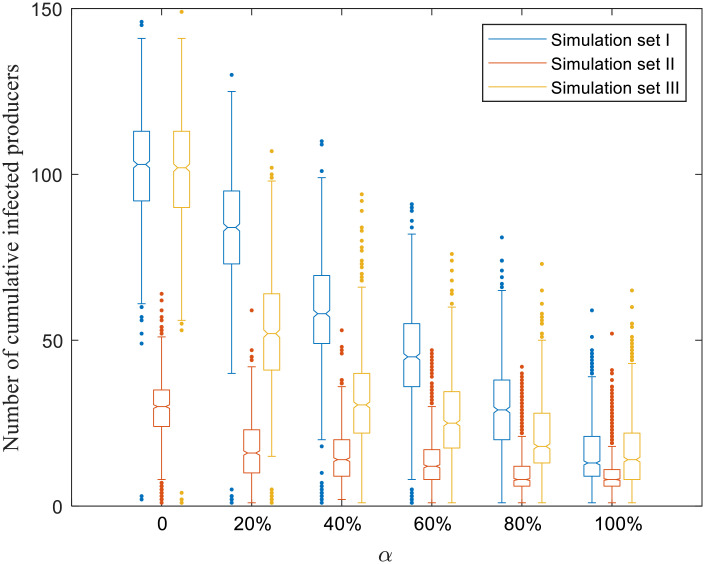
Distributions of the number of cumulative infected producers.

**Fig 5:**
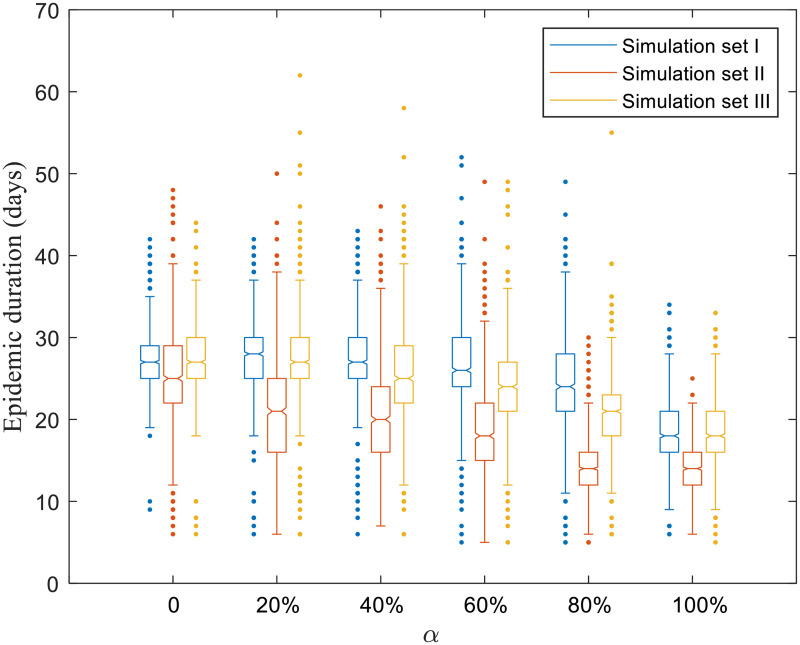
Distributions of the epidemic duration.

**Fig 6:**
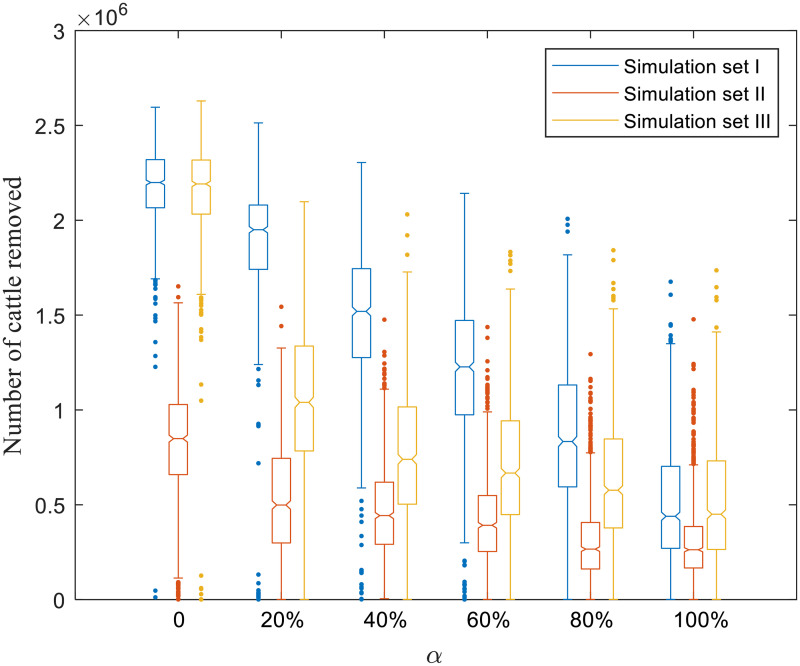
Distributions of the number of cattle removed.

**Fig 7:**
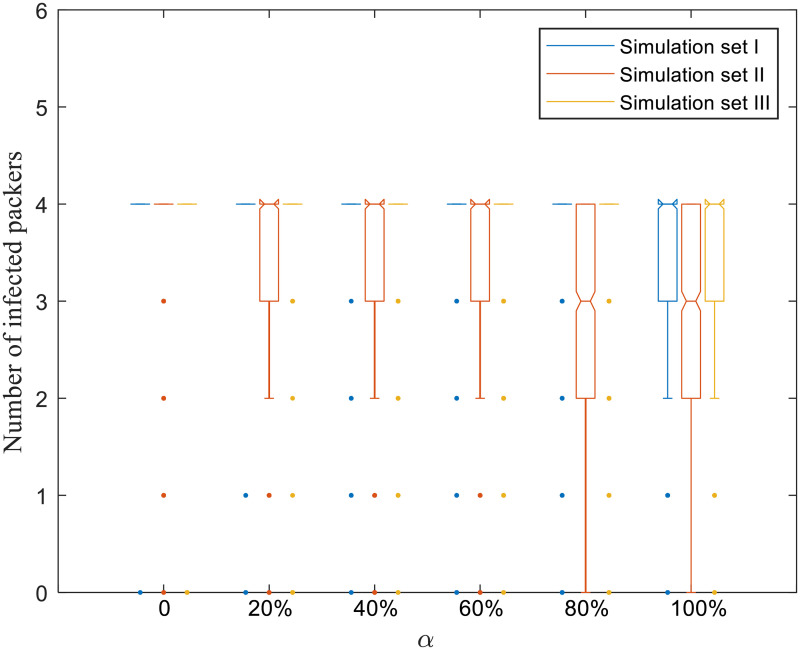
Distributions of the number of infected packers.

**Fig 8:**
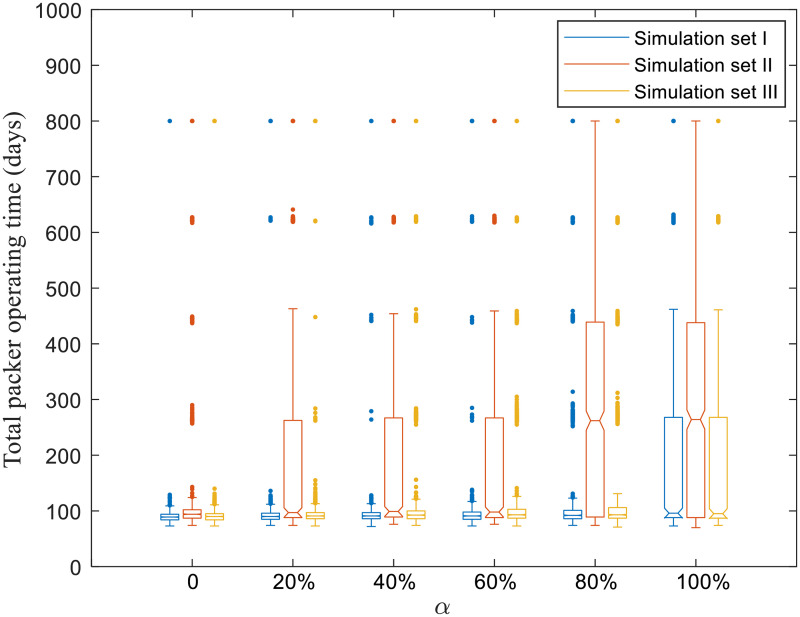
Distributions of the total packer operating time.

Figs 4–8 present boxplots of the distributions of simulation results obtained from ABM for the three simulation sets.

### 3.1 Impact of risk attitudes on producers

Fig 4 shows that increasing the percentage of producers in the risk-averse category can reduce the number of infected producers for all simulation sets. For simulation set I (blue) and *α* ranging from 0 to 100%, the median [interquartile range] number of cumulative infected farms decreases dramatically from 103 [92–113] to 13 [9–21]. Compared to simulation set I, the number of cumulative infected producers decreases much less sharply in simulation set II, which is associated with a higher initial biosecurity level. More specifically, when *α* changes from 0 to 80%, the number of cumulative infected producers changes from 30 [24–35] to 8 [6–12] and then remains almost unchanged between *α* = 80% and *α* = 100%. It indicates that when the overall biosecurity level is relatively low, changing individuals’ risk attitudes towards risk-averse can lead to a significant reduction in the epidemic size, which enhances the effectiveness of control measures.

The epidemic size for simulation set III (targeted selection) is smaller than that under simulation set I (random selection). For example, for *α* = 40%, the median number of infected producers under random selection (58 [49–70]) is almost twice that under targeted selection, which is 31 [22–40]. It indicates that risk communication based on a targeted strategy is more effective than the random strategy to contain the epidemic. A similar pattern of epidemic duration is observed in Fig 5.

### 3.2 Impact of risk attitudes on cattle losses

In the simulation, when the producer agent detects an infection, it will depopulate its cattle (all cattle transition to the removed state). Fig 6 shows the distributions of the number of cattle removed, reflecting the cattle losses.

For simulation set I, when *α* varies between 0–100%, the median [interquartile range] number of cattle removed ranges changes from 2,199,045 [2,065,838–2,319,615] to 439,215 [270,210–702,540]. For simulation set II, as *α* increases from 0–100%, cattle losses reduced from 848,640 [658,853–1,28,588] to 262,748 [166,830–385,223]. Comparing results in simulation sets I and III, we can conclude that with the same initial biosecurity distributions, targeted selection of producers to a more risk-averse behavior can yield fewer cattle losses than the random strategy. For instance, the number of cattle removed is 739,778 [502,935–1,15,755] under a targeted selection and 1,519,763 [1,275,960–1,744,928] under a random selection at *α* = 40%.

### 3.3 Impact of risk attitudes on packers

The US beef slaughter industry is heavily concentrated, with just four packers in Kansas accounting for more than 80% of the area’s total beef slaughter capacity [42]. As packers are a critical component of the food production system, we visualize the distribution characteristics across scenarios in Figs 7 and 8. As the simulation period for each run is 200 days, the maximum possible value of the total packer operating time is 800 days.

In Fig 7, the median number of infected packers is 4 for all scenarios in simulation sets I and III. For simulation set II, the median number of infected packers is 4 for *α* ≤ 60%, and becomes 3 for *α* > 60%. Similarly, in Fig 8, the median total packer operating time across all scenarios for simulation sets I and III ranges between 89–96 days and 90–95 days, respectively. For simulation set II, the range of the medium total packer operating time is 94–99 days for *α* ≤ 60%, and is 262–264 days for *α* > 60%.

In summary, the overall patterns of the indicators across scenarios are consistent over the three simulation sets. A more risk-averse population can reduce the number of infected producers/packers and cattle losses. Notably, a targeted selection of producers is more effective than the random strategy under the same initial biosecurity levels.

## 4. Conclusion

In this study, an agent-based model was developed to simulate the human decision-making process during epidemics, a factor which is often ignored in conventional livestock disease models. A case study of southwest Kansas was performed to demonstrate the model’s ability to simulate the human decision-making process around biosecurity practices as disease dynamics change. The assumptions relevant to the disease-related decision-making process enable the model to capture the complicated interactions between disease spreading and individuals’ responsiveness on biosecurity measures, considering individuals’ heterogeneous risk attitudes. The model developed can be used by policymakers to examine the effectiveness of various control measures and identify important human behavior factors related to epidemiological parameters in disease transmission dynamics.

The simulation results show that human behavioral responses to biosecurity events, operationalized as risk attitude, substantially affect the epidemic dynamics and the effectiveness of movement restrictions. Additionally, knowing the relevant stakeholders’ initial biosecurity level is vital for cost-effective risk communication. When the initial biosecurity level is relatively low, nudging producers’ risk attitudes towards greater risk-aversion can significantly reduce the cumulative number of infected producer locations. When the initial biosecurity level is high, communication to increase risk-aversion will lead to a smaller reduction in the final epidemic size. Compared to the scenario in which all producers are risk-tolerant, we observe that if about half of producers adopt a risk-averse behavior, this can lead to a sharp decrease in the median number of infected producers, assuming that producers initially have low compliance for biosecurity protocols. We also highlighted the advantage of a targeted strategy based on producers’ capacity in selecting audiences for communications that promote more risk-aversion over a random selection strategy. In terms of packer operations, there is little difference between the random and targeted strategies for a low initial biosecurity level. This implies that risk communication on producers towards risk-aversion regardless of their biosecurity status cannot effectively protect the packers. Packers are the critical components in the system, and it’s significant to ensure overall high compliance to biosecurity level in the region to minimize the risk of disease outbreaks.

## Supporting information

S1 Data Data for the three simulation sets. (ZIP)
